# Relapse Patterns and Clinical Outcomes in Cardiac Sarcoidosis: Insights from a Retrospective Single-Center Cohort Study

**DOI:** 10.3390/jcm14176234

**Published:** 2025-09-03

**Authors:** Arnaud Dominati, Geoffrey Urbanski, Philippe Meyer, Jörg D. Seebach

**Affiliations:** 1 Division of Clinical Immunology and Allergology, Department of Medicine, Geneva University Hospital, 1205 Geneva, Switzerland; 2 Division of Rheumatology, Department of Medicine, University of Illinois College of Medicine in Chicago, Chicago, IL 60612, USA; 3 Department of Orofacial Sciences, University of California San Francisco, San Francisco, CA 94143, USA; 4 Division of Cardiology, Department of Medicine, Geneva University Hospital, 1205 Geneva, Switzerland

**Keywords:** cardiac sarcoidosis, granulomatous inflammation, immunosuppressive therapy, major adverse cardiac events, positron emission tomography, relapse

## Abstract

**Background/Objectives:** Cardiac sarcoidosis (CS) is a granulomatous inflammatory cardiomyopathy with heterogeneous presentations, from palpitations to heart failure and sudden cardiac arrest. Despite advances in imaging and immunosuppressive (IS) therapy, relapse patterns and long-term outcomes remain poorly defined. This study aimed to characterize relapse and identify predictors of relapse and major adverse cardiac events (MACE) in a real-world CS cohort. **Methods:** This retrospective single-center study included 25 adults diagnosed with CS at Geneva University Hospitals between 2016 and 2024, classified per the 2024 American Heart Association diagnostic criteria. Relapse was defined as clinical, arrhythmic, or imaging deterioration requiring treatment escalation. MACE included cardiovascular hospitalization, device therapy, left ventricular assist device, heart transplant, or death. Statistical methods included Kaplan–Meier analysis with log-rank tests and multivariable Cox regression adjusted for age and sex. **Results:** Relapse occurred in 13 patients (56%), frequently subclinical (61.5%) and detected incidentally on routine PET-CT during IS tapering. In the multivariate model, predictors of relapse included right ventricular FDG uptake (aHR 13.1; 95% CI 1.3–133.7; *p* = 0.03) and second-line immunosuppression duration ≤24 months (aHR 20.1; 95% CI 1.1–363.8; *p* = 0.04). Relapse-free patients were more often maintained on dual or triple IS therapy (71.4% vs. 15.4%; *p* = 0.02) and low-dose prednisone (<10 mg/day) (57.1% vs. 7.7%; *p* = 0.03). **Conclusions:** Relapse is common in CS, often subclinical, and associated with PET-CT findings and premature IS tapering. Maintenance therapy may reduce risk. Multimodal imaging remains critical for disease monitoring, though tracers with higher specificity are needed. Further research should refine relapse definitions and support personalized treatment strategies.

## 1. Introduction

Cardiac sarcoidosis (CS) is a granulomatous inflammatory cardiomyopathy that presents with a broad spectrum of clinical manifestations, ranging from palpitations to heart failure and sudden cardiac arrest. Although clinical cardiac involvement is detected in approximately 5% of patients with systemic sarcoidosis, autopsy studies suggest a much higher prevalence, approaching 20% [1]. The widespread use of cardiac magnetic resonance imaging (cMRI) and ^18^Fluor-fluorodeoxyglucose positron emission tomography (^18^F-FDG PET-CT) has markedly improved the detection of myocardial abnormalities suggestive of CS [2], including cases of isolated CS [3], where cardiac involvement is present without extracardiac disease. In one notable study, nearly one third of middle-aged patients presenting with high-grade atrioventricular block (AVB), previously considered idiopathic, were found to have CS. All of these patients were male and more often exhibited heart failure symptoms, increased interventricular septal thickness, and right ventricular dysfunction compared with those whose AVB remained idiopathic [4].

Despite advances in diagnostic techniques, CS remains a challenging condition to identify and is the second leading cause of sarcoidosis-related mortality, following pulmonary involvement [5,6,7,8]. However, recent data from a nationwide Finnish cohort showed a substantial increase in CS incidence between 1985 and 2020, likely due to improved clinical awareness and the broader adoption of advanced cardiac imaging modalities [9]. This increase coincided with a trend toward milder clinical presentations at the time of diagnosis and improved long-term outcomes, suggesting that earlier detection and evolving management strategies may be contributing to a more favorable prognosis in CS.

Management of CS is multifaceted, combining immunosuppressive (IS) therapy with guideline-directed treatment for heart failure and arrhythmia control. IS therapy plays a central role in controlling myocardial inflammation and is endorsed by consensus guidelines [10,11]. However, recommendations regarding corticosteroid dosing, treatment duration, and the optimal use of steroid-sparing agents remain largely based on observational data and expert opinion rather than randomized controlled trials. Consequently, treatment practices often diverge based on disease severity, institutional protocols, and clinical experience.

One of the most pressing challenges in long-term management of CS is the high rate of relapse, which may manifest overtly, through clinical deterioration, or remain entirely subclinical, detectable only via routine imaging surveillance [12,13,14]. Relapse contributes to cumulative myocardial injury and adds complexity to therapeutic decision-making. Despite its clinical significance, the underlying risk factors and reliable predictors of relapse remain poorly defined, underscoring the need for further research to guide individualized monitoring and treatment strategies. Improving our understanding of relapse dynamics and associated outcomes is essential for optimizing long-term care in CS.

The present retrospective study aimed to investigate the occurrence of cardiac relapse and identify potential clinical, imaging, and treatment-related predictors. A secondary objective was to evaluate the rate of major adverse cardiac events (MACE) and to explore associated risk factors.

## 2. Materials and Methods

### 2.1. Ethics

The study was conducted in accordance with the Declaration of Helsinki and approved by the Cantonal Ethics Committee for Research Involving Human Subjects of Geneva (Swissethics, BASEC number 2024-01883; date of approval: 26 November 2024). Written general consent for retrospective research was obtained from all living patients in accordance with institutional guidelines. For deceased patients, the ethics committee granted an exemption from consent in cases where no objection to the use of medical data for research purposes was documented in the medical record.

### 2.2. Study Design and Population

This retrospective single-center study was conducted at the Division of Clinical Immunology and Allergology, Geneva University Hospitals (HUG). All consecutive adult patients (≥18 years) diagnosed with CS between January 2016 and December 2024 were included. Inclusion required a diagnosis of CS classified as definite, highly probable, or probable, according to the diagnostic algorithm proposed by the 2024 American Heart Association (AHA) [11]. This algorithm is based on the 2014 Heart Rhythm Society (HRS) [15] and the 2016 Japanese Circulation Society (JCS) criteria [16]. Patients were excluded if they did not meet diagnostic criteria or had formally declined the use of their data.

Clinical data were extracted from the institutional electronic health record system (Dossier Patient Informatisé—DPI), including baseline characteristics, diagnostic workup, IS treatments, and long-term outcomes.

The primary outcome was relapse of CS, defined as clinical, arrhythmic, or imaging deterioration requiring treatment escalation. Subclinical relapse was further specified as new FDG uptake on PET-CT without concomitant cardiac symptoms or arrhythmias. For descriptive analyses, patients in the non-relapse group (NRG) were required to have at least twelve months of follow-up, documented resolution of the initial episode, and a minimum of six months of follow-up after resolution, leaving seven patients for descriptive comparison with the thirteen patients who relapsed. For time-to-event analyses, all patients were included, regardless of follow-up duration, as Cox regression inherently accounts for differences in follow-up through censoring.

The secondary outcome was major adverse cardiac events (MACE), defined as a composite of unplanned cardiovascular hospitalizations, appropriate implantable cardioverter–defibrillator (ICD) shocks, left ventricular assist device (LVAD) implantation, heart transplantation, or all-cause mortality.

Ventricular arrhythmias (VA) were defined as ≥1% premature ventricular contractions (PVCs), non-sustained or sustained ventricular tachycardia (VT), ventricular fibrillation (VF), or accelerated idioventricular rhythm (AIVR).

### 2.3. Statistical Analysis

Descriptive statistics were reported as medians with interquartile ranges for continuous variables, and absolute values with percentages for categorical variables. Comparisons between the relapse group (RG) and NRG, as well as between patients with and without MACE, were performed using Fisher’s exact test for categorical variables and the Mann–Whitney U test for continuous variables. Kaplan–Meier survival curves and log-rank tests were used to compare relapse-free and MACE-free survival between groups. Cox proportional hazards regression was used to identify predictors of relapse and MACE. Variables with a *p*-value < 0.1 in univariate analysis were further evaluated in multivariate models adjusted for age and sex. Multicollinearity was assessed using variance inflation factors. The proportional hazards assumption was verified using Schoenfeld residuals, and the linearity of continuous variables was assessed using Martingale residual plots. Adjusted hazard ratios (aHR) with 95% confidence intervals (CI) and corresponding *p*-values were reported.

All statistical analyses were conducted in R version 4.4.1 (R Foundation for Statistical Computing, Vienna, Austria), using the survival 3.7-0, dplyr 1.1.4, broom 1.0.7, car 3.1-3, survminer 0.4.9, and logistf 1.26.1 packages.

## 3. Results

### 3.1. Baseline Characteristics, Diagnostic Findings, and Initial Treatment Strategies

The cohort included 25 patients with CS, predominantly male (76%) and Caucasian (92%). Median age at diagnosis was 53 years (47.0–61.0), and the median follow-up period was 56 months (42.0–84.5). CS represented the initial manifestation of sarcoidosis in 13 cases (52%), and isolated cardiac involvement was observed in three patients (12%). General characteristics are summarized in Table 1, while details of extracardiac organ involvement are presented in Supplementary Table S1, and all laboratory findings are reported in Supplementary Table S2.

Biopsy-confirmed sarcoidosis was obtained in 21 patients (84%). Endomyocardial biopsy was performed in seven patients (28%), revealing non-necrotizing granulomas in four (57.1%), lymphocytic inflammation without fibrosis in one, subendocardial fibrosis without inflammation in another one, and no abnormalities in the last one.

**Figure 1:**
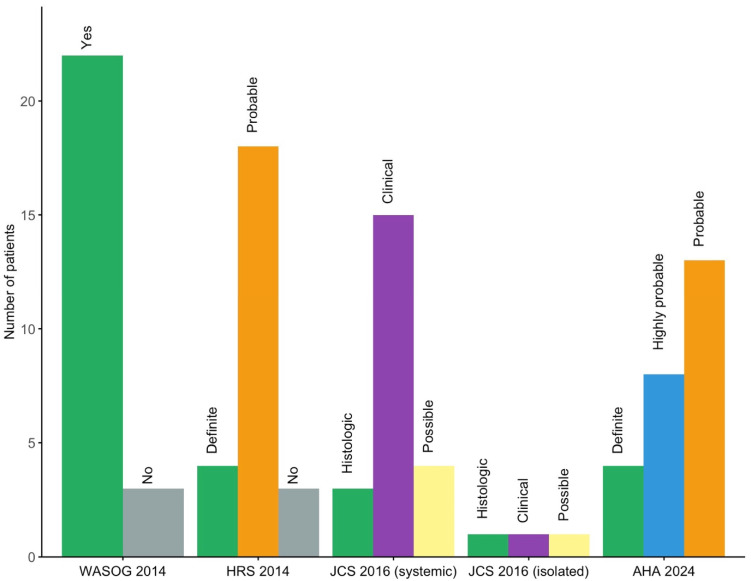
Distribution of patients across cardiac sarcoidosis diagnostic criteria (2014 WASOG, 2014 HRS, 2016 JCS, 2024 AHA). AHA: American Heart Association; HRS: Heart Rhythm Society; JCS: Japanese Circulation Society; WASOG: World Association of Sarcoidosis and Other Granulomatous Diseases.

According to the 2024 AHA diagnostic algorithm, four patients (16%) were classified as definite CS, eight (32%) as highly probable, and thirteen (52%) as probable. Figure 1 presents a comparison of the 2014 WASOG, 2014 HRS, 2016 JCS, and 2024 AHA diagnostic criteria, highlighting differences in the classification of CS across these systems.

**Figure 2:**
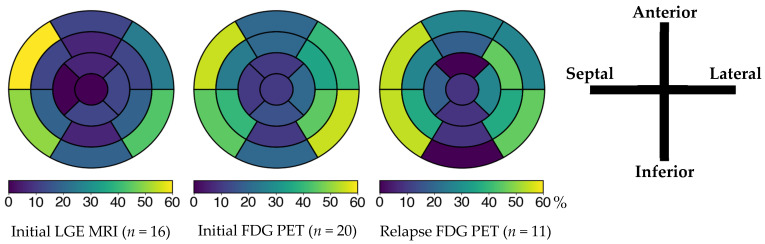
Left ventricular involvement on cardiac MRI and ^18^F-FDG PET according to the AHA 17-segment model at initial episode and relapse. The color bar below the AHA 17-segment model indicates the percentage of segment involvement, with color intensity corresponding to the proportion of patients showing abnormal findings in each segment. In all three assessments, the basal anteroseptal, inferoseptal, and inferolateral segments were the most involved. FDG: fluorodeoxyglucose; LGE: late gadolinium enhancement; MRI: magnetic resonance imaging; PET: positron emission tomography.

**Figure 3:**
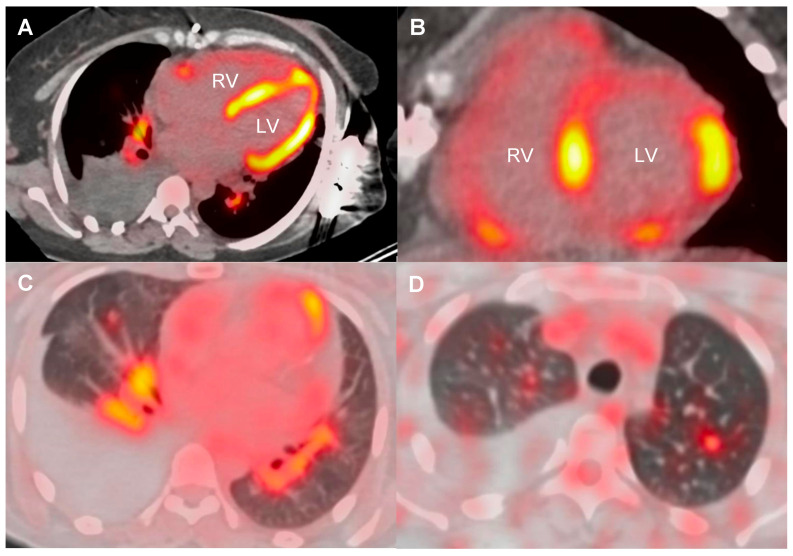
^18^F-FDG PET-CT showing cardiac, hilar lymph node, and lung involvement in sarcoidosis. Axial PET-CT images showing ^18^F-FDG uptake in both ventricles (**A**,**B**), hilar lymph nodes (**A**,**C**), and lung parenchyma (**C**,**D**), consistent with multiorgan sarcoidosis. LV: left ventricle; RV: right ventricle.

At diagnosis, 6 patients (24%) had left ventricular ejection fraction (LVEF) below 50%, nine (36%) had high-grade AVB, and 13 (52%) had VA. Four (16%) had a prior history of arrhythmias initially classified as idiopathic, with time to diagnosis extending up to 11 years. Additional baseline features, including advanced imaging and laboratory findings, are summarized in Table 2. All ECG, Holter, and device monitoring findings at initial presentation, during follow-up, and at last follow-up are detailed in Supplementary Table S3. ^18^F-FDG PET-CT showed abnormal left ventricular uptake in 20 of 22 cases (91%), most frequently involving the basal septum and the basal inferolateral segment (Figure 2). Extracardiac FDG uptake was present in 16 of 20 patients (80%) (Figure 3).

First-line treatment included prednisone in 22 patients (88%), with a median starting dose of 0.6 mg/kg/day (0.50–0.82). The median treatment duration was 19 months (11.0–26.2), with tapering below 10 mg/day occurring after a median of 4.5 months (3.2–5.5; *n* = 16). Second-line agents were used in 24 patients (96%), most commonly subcutaneous methotrexate (68%), followed by azathioprine (20%) and mycophenolate mofetil (8%) (Table 3). Anti-TNF agents were prescribed as third-line therapy in seven patients (28%): five received infliximab and two adalimumab. At last follow-up, six patients (including two on adalimumab) remained on anti-TNF therapy, all showing complete resolution of CS. Among them, three continued low-dose prednisone (<10 mg/day).

**Figure 4:**
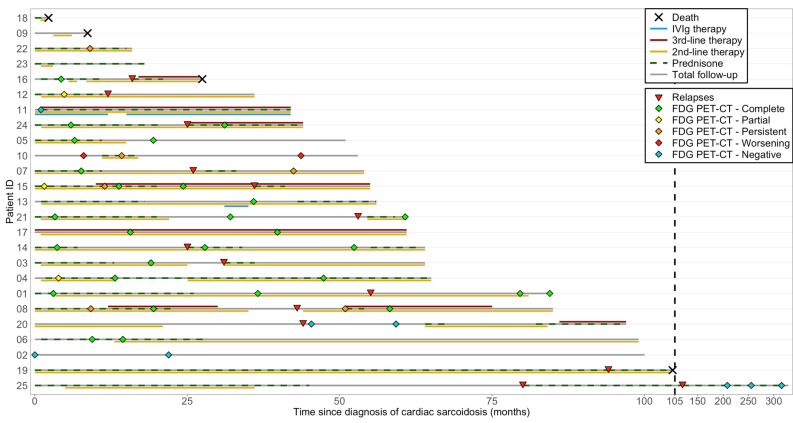
Swimmer plot of individual longitudinal trajectories of treatment and outcomes in cardiac sarcoidosis. This swimmer plot illustrates the longitudinal follow-up of patients with cardiac sarcoidosis, including total follow-up duration, immunosuppressive therapies, PET-CT responses, relapses, and deaths. PET-CT responses are labeled as “FDG PET-CT—complete,” “partial,” “persistent,” and “worsening,” which correspond to the evolution of uptake in patients who initially had myocardial FDG uptake prior to IS treatment initiation. “FDG PET-CT—negative” indicates absence of FDG uptake before therapy. Treatment bars show the duration (not dosage) of immunosuppressive therapies. Second-line therapy includes methotrexate, azathioprine, mycophenolate mofetil, and sirolimus (rapamycin), while third-line therapy includes adalimumab and infliximab. Some details regarding individual patient management are provided below when deemed relevant. Patient 1 presented with many non-sustained VT on last device monitoring with negative FDG PET-CT. Relapse 1 showed slight new focal uptake but no symptoms or VA, with normal LVEF, and methotrexate was increased from 7.5 to 20 mg/week. Patient 2 had biopsy-proven extracardiac sarcoidosis and presented with high-grade AVB and frequent premature ventricular contractions, but without left ventricular dysfunction, contractility abnormalities, or any abnormalities on PET-CT or cardiac MRI. Patient 3 had no PET-CT after relapse but maintained normal LVEF and had no documented arrhythmia at last follow-up. Patient 10 was treated with prednisone and methotrexate for six months, but discontinued therapy due to a lack of improvement on follow-up PET-CT. Since then, the patient has developed progressive left ventricular hypertrophy, without systolic dysfunction or contractility abnormalities. A comprehensive genetic panel did not reveal any pathogenic or likely pathogenic variant. However, a variant of uncertain clinical significance was identified in the MYH7 gene. Patient 11 was treated with IVIg for sarcoidosis-related inflammatory peripheral polyneuropathy and relapsed after attempting to stop therapy. Patient 12 did not undergo PET-CT after relapse but had preserved LVEF and no VA or high-grade AVB recurrence. Patient 13 received IVIg for inclusion body myositis. Patient 14 received prednisone (20 mg) at the end of follow-up for an asymptomatic lung relapse confirmed by a positive biopsy. Patient 20 had two courses of prednisone during follow-up for extra-CS relapses. Patient 23 had stable LGE since CS diagnosis on last cMRI and resolution of premature ventricular contractions. Patient 25 had two relapses (no PET-CT performed) marked by increased arrhythmias treated with high-dose prednisone. AVB: atrioventricular block; FDG: fluorodeoxyglucose; IVIg: intravenous immunoglobulin; PET-CT: positron emission tomography–computed tomography; LVEF: left ventricular ejection fraction; MRI: magnetic resonance imaging; LGE: late gadolinium enhancement; VA: ventricular arrhythmias.

Adverse events related to IS agents and implanted devices were not uncommon, and infections occurred in a subset of patients during follow-up (Table 4). Treatment courses, PET-CT results, and key clinical events, including relapse and death, are illustrated in a swimmer plot (Figure 4).

### 3.2. Arrhythmias and Imaging Follow-Up

Over a median follow-up of 56 months (42.0–84.5), VA occurred in 20 of 23 monitored patients (87%). Of thirteen patients with recent device or rhythm monitoring data, ten (77%) had no residual VA, two improved, and one was stable. Anti-arrhythmic drugs were used in 14 (56%) patients.

Of the eight patients with high-grade AVB at presentation, one underwent heart transplantation and six received IS therapy, of whom two (33.3%) experienced resolution of conduction abnormalities.

Among 22 evaluable patients (excluding two early deaths and one transplant), there was no significant change in LVEF between diagnosis and serial follow-up timepoints at six, 12, 24, and 60 months, or at last evaluation (Supplementary Table S4).

PET-CT evolution mirrored clinical improvement in most cases. Among the 17 patients with post-treatment PET-CT after the initial episode, 14 (82%) showed complete resolution, 1 (6%) partial resolution, and 2 (12%) persistent but non-progressive uptake. Median time from initial to follow-up PET-CT was 9.0 months (4.3–14.2). Among the five patients with post-relapse PET-CT, four (80%) showed resolution, and one remained stable. Median time from relapse to PET-CT was 7.8 months (6.1–16.4).

Necrotic lesions were identified in six patients (24%) during follow-up, compared to only one (4%) at baseline. Follow-up cMRI (*n* = 8) performed after a median of 13.2 months (7.8–17.5) revealed persistent LGE in five patients (62.5%) and partial resolution in three (37.5%), while none showed complete resolution.

### 3.3. Major Adverse Cardiac Events and Their Potential Predictors

**Figure 5:**
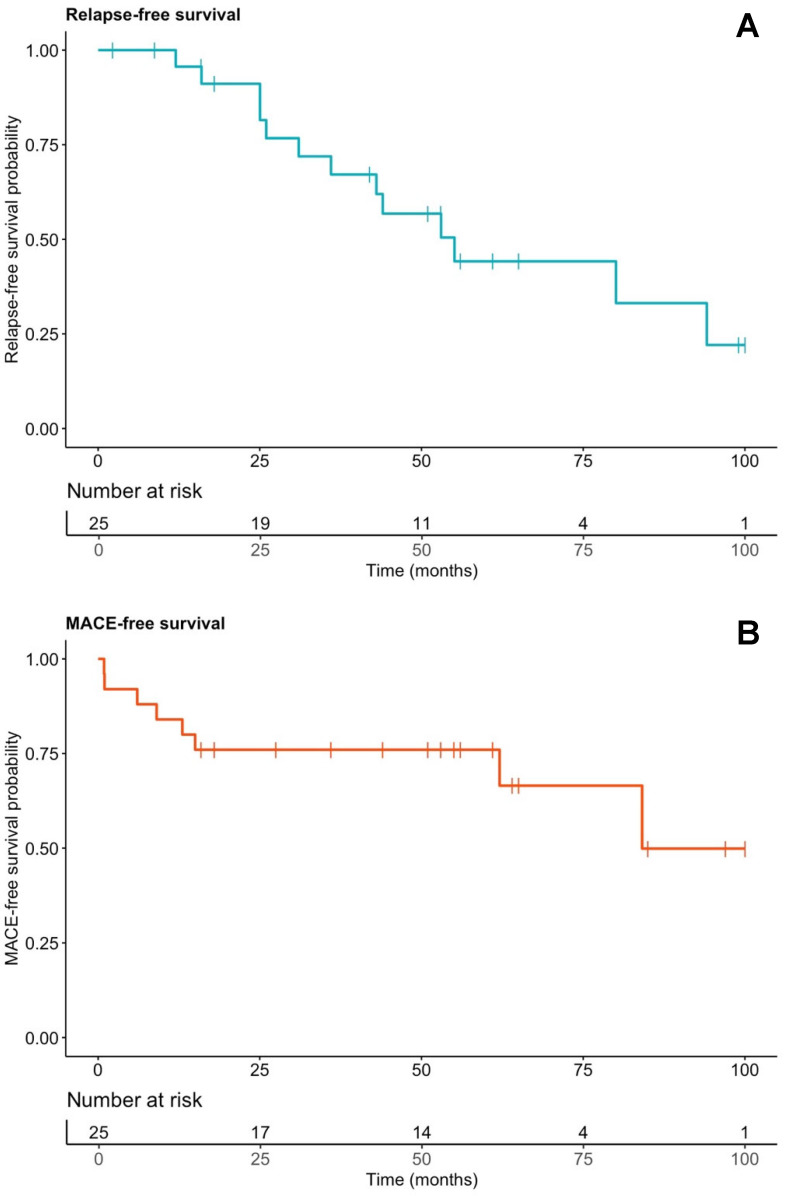
Kaplan–Meier curves of relapse-free (**A**) and MACE-free (**B**) survival in the whole cohort. Vertical bars represent censored events. MACE: major adverse cardiac events.

Nine patients (36.0%) experienced at least one MACE (Table 5). One patient underwent orthotopic heart transplantation and remained relapse-free for 6.7 years under continued IS therapy to prevent graft rejection. Four patients (16.0%) died during follow-up, including two CS-related deaths: one from sudden cardiac death secondary to VF and one from end-stage heart failure after delayed diagnosis. At 48 months, 24% had experienced at least one MACE (Figure 5B). Comparisons by MACE status are presented in Supplementary Table S5.

Three predictors, definite CS diagnosis, LVEF < 50% with VA, and left ventricular LGE involvement of ≥5 segments, were significant in univariate analysis (Supplementary Table S6). LGE extent lost significance in adjusted models for age and sex, but both definite CS diagnosis (aHR 6.5, 95% CI 1.53–27.3; *p* = 0.01) and LVEF < 50% with VA (aHR 10.3, 95% CI 1.1–94.4; *p* = 0.04) remained independently associated with MACE. Due to substantial multicollinearity between these two variables, only LVEF < 50% with VA and left ventricular LGE ≥ 5 segments were retained in the multivariate model, adjusted for age and sex. Neither variable was significantly associated with MACE, likely due to limited power.

### 3.4. Relapse Characteristics and Their Potential Predictors

Fourteen cardiac relapses occurred in thirteen of twenty-five patients (56%). By 48 months, 43.2% had experienced at least one relapse (Figure 5A). First relapse occurred at a median of 33.6 months (25.0–53.0) after diagnosis. Relapse was asymptomatic and detected on PET-CT during IS tapering in eight patients (61.5%); the remainder (38.5%) had recurrent arrhythmias, with or without symptoms (Table 6). At relapse, eight patients (61.5%) were still on IS therapy, mostly methotrexate (*n* = 6). In these patients, relapse occurred at significantly lower methotrexate doses compared to the levels before tapering was initiated (0.15 [0.12–0.18] vs. 0.23 [0.19–0.25] mg/kg/week; *p* = 0.03), suggesting that tapering below a certain threshold may contribute to disease reactivation. In the remaining five patients (38.5%), IS had been discontinued for a median of 23 months (8–31) before relapse.

Following relapse diagnosis, most patients resumed corticosteroids. Compared to initial therapy, prednisone was restarted at lower doses, tapered more quickly, and used for a shorter duration (Table 7).

Extracardiac relapses occurred in three patients (12%), accounting for five episodes, including three in a single individual.

#### Potential Predictors of Relapse

Treatment at relapse in the RG was compared to treatment at last follow-up in the NRG. Use of dual or triple IS therapy was significantly more frequent in the NRG compared to RG (71.4% vs. 15.4%; *p* = 0.02), as was prednisone use (57.1% vs. 7.7%; *p* = 0.03).

In univariate Cox regression analysis, three variables, right ventricular FDG uptake, initial prednisone duration ≤18 months, and second-line IS duration ≤24 months, were significant. In multivariate analysis adjusted for age and sex, both right ventricular FDG uptake (aHR 13.1, 95% CI 1.3–133.7; *p* = 0.03) and second-line IS duration ≤24 months (aHR 20.1, 95% CI 1.1–363.8; *p* = 0.04) were independently associated with relapse. Prednisone duration ≤18 months lost significance in the full model.

## 4. Discussion

CS remains a complex and heterogeneous condition, presenting ongoing challenges in diagnosis, risk stratification, and long-term management. Although advances in imaging modalities and IS strategies have improved initial disease control, relapse is frequent, and optimal approaches to treatment tapering or discontinuation are yet to be clearly defined. This study identifies right ventricular FDG uptake and duration of IS second-line therapy ≤24 months as potential risk factors for relapse, underscoring the need for sustained IS in selected patients.

This retrospective study of 25 patients with CS, followed over a median of 56 months, revealed a relapse rate exceeding 50%, with many episodes identified asymptomatically through routine ^18^F-FDG PET-CT surveillance. Although 82% of patients achieved complete metabolic response after initial treatment, long-term control of inflammation remained elusive. Structural disease progression was also observed, with persistent LGE and increased detection of necrotic myocardial lesions on follow-up imaging, suggesting ongoing cardiac remodeling despite apparent clinical and metabolic improvement. Baseline imaging showed strong concordance between ^18^F-FDG PET-CT and cMRI, with the basal anteroseptal and inferoseptal segments being the most frequently affected, findings that are consistent with previous reports [17,18]. These observations underscore the importance of multimodal imaging in both initial assessment and longitudinal monitoring and highlight the need for refined strategies to sustain remission and prevent subclinical disease progression.

This cohort demonstrated the wide clinical spectrum of CS in a tertiary care setting, with frequent initial presentations including conduction abnormalities, VA, and reduced LVEF. In some patients with known systemic sarcoidosis, CS was diagnosed decades later, highlighting that cardiac involvement may occur long after initial disease onset. Diagnostic delays were not uncommon, with CS identified up to 11 years after unexplained arrhythmias, highlighting the need for heightened clinical suspicion. Indeed, in a retrospective study of 30 unexplained high-grade AVB, 33% were ultimately attributed to CS via advanced imaging [4]. Another study of 77 patients with CS and high-grade AVB reported a median delay of 112 days between AVB onset and CS diagnosis. Patients diagnosed later experienced more adverse events, more frequent device upgrades, and required higher maintenance steroid doses [19], suggesting that early cardiac evaluation with advanced imaging may contribute to improved outcomes.

Classification criteria remain a source of variability, as reflected by divergent diagnoses under the 2014 WASOG, 2014 HRS, 2016 JCS, and 2024 AHA criteria (Figure 1). Notably, the 2016 JCS criteria permit diagnosis based solely on clinical and imaging data, including for isolated CS, without requiring histologic confirmation. In contrast, the WASOG and HRS frameworks require biopsy-proven extracardiac sarcoidosis for a probable CS diagnosis. These discrepancies influence diagnostic sensitivity and specificity, and none of these systems has yet undergone comprehensive external validation [20]. Recently, in the CHASM-CS cohort (NCT01477359), the HRS criteria showed modest sensitivity (47.7%) but high specificity (89.4%). A modified approach substituting histological confirmation with suggestive CT or PET-CT findings improved sensitivity to 97.7%, though specificity declined to 81.6% [21]. However, no direct comparison has been made between modified HRS, JCS, and AHA criteria, and isolated CS remains difficult to diagnose without histology. In this study, patients classified as “definite” CS experienced significantly more MACE, consistent with prior reports, likely reflecting greater severity and prompting endomyocardial biopsy [22].

Almost all patients received corticosteroids along with a second-line agent, most commonly methotrexate. The early introduction of combination therapy likely reflected heightened clinical awareness of relapse risk, prompting a proactive treatment strategy [12,13,14,23]. In a prospective trial using a 12-month prednisone taper, subclinical relapse occurred in 12 of 19 patients (63.1%) within three months of stopping therapy [24]. A retrospective study showed higher relapse rates among patients treated with corticosteroids alone compared to combination therapy, underscoring the potential benefit of adjunctive IS in sustaining disease control [25].

In our cohort, relapse was common, with many episodes detected subclinically through routine imaging surveillance. Despite the absence of overt symptoms, these relapses prompted treatment intensification to prevent potential clinical deterioration, arrhythmias, or progressive ventricular dysfunction. A similar pattern was reported in a Japanese retrospective study, where subclinical relapse frequently led to treatment escalation [13]. However, no significant difference in MACE was observed compared to patients without relapse. Whether this reflects the benign nature of subclinical relapse or the benefit of early intervention remains uncertain. Another study emphasized the prognostic implications of subclinical disease activity by evaluating the use of iodine-123 β-methyl iodophenyl pentadecanoic acid (BMIPP) SPECT in patients with CS treated with prednisolone [26]. BMIPP was used to assess myocardial damage through a defect score reflecting impaired fatty acid metabolism. This score was significantly higher among patients who experienced relapse and was also associated with an increased risk of MACE. In contrast to our data, a recent prospective study evaluating methotrexate withdrawal in 12 patients with CS reported only one cardiac relapse (8.3%) after a mean post-cessation follow-up of 24.9 ± 12.3 months [27]. However, the last follow-up PET-CTs were conducted after a mean of 10.6 ± 8.8 months, potentially missing later-onset subclinical relapses, the predominant pattern in our cohort. Altogether, these findings underscore the need to better understand the clinical significance of subclinical relapse and to clarify optimal management strategies. They also highlight the importance of defining effective post-remission surveillance approaches. While clinical follow-up, including symptom assessment, ECG, Holter monitoring or device interrogation, and echocardiography, remains the cornerstone of surveillance, the integration of serial imaging may enhance detection of subclinical disease activity. This approach could play a pivotal role in the early identification of inflammatory relapse and contribute to the prevention of MACE by enabling timely therapeutic adjustments.

Several studies have identified potential predictors of relapse in CS. A U.S. cohort linked LGE burden of 11% or more and isolated CS to increased relapse risk [23]. In a large French cohort, kidney involvement, left heart failure, and wall motion abnormalities were independently associated with relapse, while skin involvement and intravenous cyclophosphamide therapy appeared protective [12]. Importantly, a separate retrospective study found no significant association between relapse and adverse cardiac outcomes [13].

In our cohort, patients who remained relapse-free were more likely to have received dual or triple IS agents and to be maintained on low-dose prednisone at last follow-up, compared to those who relapsed. In addition, multivariate Cox regression identified right ventricular FDG uptake on baseline PET-CT and second-line IS duration ≤24 months as independent predictors of relapse. These findings suggest that sustained maintenance therapy may help reduce relapse risk in selected patients. However, prolonged IS is not universally required, underscoring the importance of individualized risk stratification to optimize treatment duration and avoid overtreatment. The association between right ventricular FDG uptake and relapse risk is particularly compelling. While this imaging feature has previously been linked to adverse outcomes such as VA [22,28], its role as a predictor of relapse represents a potentially novel prognostic marker that warrants validation in larger prospective cohorts. A possible explanation is that right ventricular involvement reflects a more diffuse or extensive inflammatory burden, predisposing patients to recurrent disease activity.

MACE occurred in 36% of patients, including four deaths (two directly attributable to CS) and one heart transplant. In a model adjusted for age and sex, both a definite CS diagnosis and the combination of LVEF < 50% with VA remained independently associated with MACE, consistent with previous reports [22]. LGE involving ≥5 segments was significantly associated with adverse outcomes in univariate analysis, but lost significance in adjusted models. Nevertheless, the extent of LGE has been consistently validated in prior studies as a robust predictor of poor prognosis in cardiac sarcoidosis [29,30,31,32,33,34,35,36,37]. Its limited significance in this cohort is likely attributable to sample size constraints, rather than lack of clinical relevance.

This study is constrained by its retrospective, single-center design and a predominance of Caucasian patients, which may limit generalizability and introduce referral bias. As a real-world analysis, treatment strategies, tapering schedules, and imaging follow-up were not standardized but instead reflected individualized clinical decisions, potentially introducing variability in relapse detection. FDG PET-CT was not systematically repeated at fixed intervals, which may have led to under- or overestimation of relapse timing. The relatively small sample size limited statistical power, particularly for multivariate analyses, and precluded validation of identified predictors in independent cohorts. Moreover, the respective contributions of IS versus anti-arrhythmic therapy, and the roles of active inflammation versus myocardial fibrosis, could not be disentangled in the context of arrhythmia management. Finally, the associations identified, such as the link between right ventricular FDG uptake and relapse, require external validation in larger, prospective, multicenter studies.

While several predictors of poor outcomes in CS have been identified [22], relapse risk stratification remains poorly defined. To address this gap, future studies should focus on refining the definition and classification of relapse by distinguishing between two distinct forms: clinical relapse, characterized by symptoms, functional decline, or arrhythmic events; and subclinical relapse, identified through PET-CT in the absence of overt clinical manifestations. Establishing such a framework could enable more tailored post-remission monitoring strategies, balancing symptom-driven clinical surveillance with imaging-guided follow-up. Ultimately, the identification of reliable relapse predictors will be essential to improve long-term outcomes.

## 5. Conclusions

Relapse in CS is common, often subclinical, and typically occurs during or following tapering of IS therapy. Although extended treatment might mitigate the risk of recurrence, it must be carefully weighed against the potential risk of cumulative toxicity. Multimodal imaging, particularly ^18^F-FDG PET-CT and cMRI, remains indispensable for diagnosis, longitudinal monitoring, and relapse detection. While right ventricular FDG uptake has been associated with adverse prognosis, our findings indicate that it may also predict relapse. Future studies should validate robust predictors of relapse, refine treatment duration and intensity through personalized therapeutic strategies, and determine the impact of relapse on long-term outcomes such as MACE.

## Figures and Tables

**Table 1:** Demographic characteristics and comorbidities in the whole cohort, and stratified by relapse status.

Variables *n* (%) or Median (IQR)	Whole Cohort (*n* = 25)	Relapse Group ^a^ (*n* = 13)	Non-Relapse Group ^a^ (*n* = 7)
Age at diagnosis of CS (years)	53.0 (47.0–61.0)	53.0 (47.0–61.0)	55.0 (51.5–65.0)
Follow-up since diagnosis of CS (months)	56.0 (42.0–84.5)	64.0 (54.0–85.0)	56.0 (46.5–63.0)
Male	19 (76.0%)	11 (84.6%)	6 (85.7%)
Caucasian	23 (92.0%)	12 (92.3%)	7 (100.0%)
Smoking habit			
Never	16 (64.0%)	7 (53.8%)	4 (57.1%)
Former	8 (32.0%)	5 (38.5%)	3 (42.9%)
BMI (kg/m^2^)	26.0 (25.2–31.4)	26.1 (24.6–28.7)	25.8 (25.5–33.6)
Comorbidities			
Dyslipidemia	9 (36.0%)	3 (23.1%)	4 (57.1%)
Diabetes	2 (8.0%)	0 (0.0%)	1 (14.3%)
Hypertension	11 (44.0%)	5 (38.5%)	5 (71.4%)
Sleep apnea syndrome	5 (20.0%)	3 (23.1%)	1 (14.3%)
Ischemic cardiomyopathy	1 (4.0%)	0 (0.0%)	1 (14.3%)
Chronic kidney insufficiency	5 (20.0%)	1 (7.7%)	2 (28.6%)
Latent or prior tuberculosis	3 (12.0%)	1 (7.7%)	1 (14.3%)
Associated autoimmune disease	5 (20.0%)	2 (15.4%)	2 (28.6%)
Sjögren’s disease	1 (4.0%)	0 (0.0%)	1 (14.3%)
Inclusion body myositis	1 (4.0%)	0 (0.0%)	1 (14.3%)
Ulcerative colitis	1 (4.0%)	0 (0.0%)	1 (14.3%)
MGUS	3 (12.0%)	2 (15.4%)	0 (0.0%)
History of malignancy ^b^	3 (12.0%)	1 (7.7%)	2 (28.6%)

BMI: body mass index; CS: cardiac sarcoidosis; IQR: interquartile range; MGUS: monoclonal gammopathy of undetermined significance. ^a^ Of the 25 patients in the overall cohort, 20 were evaluable for descriptive analysis of relapse. Five patients were excluded: Patient 2 (no immunosuppressive therapy), Patients 9 and 18 (death before minimum required follow-up), and Patients 10 and 22 (no documented resolution of the first episode on PET-CT). This resulted in 13 patients in the relapse group (RG) and 7 in the non-relapse group (NRG). ^b^ History of malignancy includes one mucosa-associated lymphoid tissue lymphoma, one prostate adenocarcinoma, and one vocal cord epidermoid carcinoma.

**Table 2:** Cardiac sarcoidosis features and initial presentation at diagnosis in the whole cohort, and stratified by relapse status.

Variables *n* (%) or Median (IQR)	Whole Cohort (*n* = 25)	Relapse Group (*n* = 13)	Non-Relapse Group (*n* = 7)
**Cardiac sarcoidosis features**			
Arrhythmia considered idiopathic before diagnosis of CS	4 (16.0%)	2 (15.4%)	2 (28.6%)
Time between idiopathic arrhythmia and diagnosis of CS (months)	96.5 (72.6–114.0); *n* = 4	96.5 (90.8–102.3); *n* = 2	83.6 (59.3–107.9); *n* = 2
CS diagnosed as initial sarcoidosis presentation	13 (52%)	8 (61.5%)	3 (42.9%)
Biopsy-proven sarcoidosis			
Cardiac	4 (16.0%)	2 (15.4%)	1 (14.3%)
Extracardiac	18 (72.0%)	10 (76.9%)	5 (71.4%)
No biopsies	3 (12.0%)	1 (7.7%)	1 (14.3%)
Diagnostic criteria—AHA likelihood Algorithm [11]			
Definite	4 (16.0%)	2 (15.4%)	1 (14.3%)
Highly probable	8 (32.0%)	6 (46.2%)	1 (14.3%)
Probable	13 (52.0%)	5 (38.5%)	5 (71.4%)
Isolated CS	3 (12.0%)	1 (7.7%)	1 (14.3%)
Cardiac relapses	14 (56.0%)	13 (100.0%)	0 (0.0%)
Extracardiac relapses	3 (12.0%)	2 (15.4%)	1 (14.3%)
**Initial presentation of cardiac sarcoidosis**			
Symptoms			
Palpitations	10 (40.0%)	7 (53.8%)	3 (42.9%)
Syncope	2 (8.0%)	1 (7.7%)	0 (0.0%)
Dizziness	6 (24.0%)	4 (30.8%)	1 (14.3%)
Chest pain	4 (16.0%)	2 (15.4%)	1 (14.3%)
Shortness of breath	11 (44.0%)	5 (38.5%)	3 (42.9%)
Clinical heart failure	4 (16.0%)	1 (7.7%)	2 (28.6%)
No cardiac symptoms	4 (16.0%)	1 (7.7%)	2 (28.6%)
Laboratory values			
Lymphocytes < 1.5 10^9^/L	9/18 (50.0%)	6/8 (75.0%)	2/6 (33.3%)
CRP > 5 mg/L	3/20 (15.0%)	2/9 (22.2%)	1/6 (16.7%)
eGFR (CKD-EPI)	86.0 (74.0–92.0)	86 (78–89.5)	78 (62.5–101)
<60 mL/min/1.73 m^2^	5/23 (21.7%)	1/11 (9.1%)	2/7 (28.6%)
ACE > 70 U/L	4/16 (25.0%)	1/7 (14.3%)	0/5 (0.0%)
High-sensitive troponin T ≥ 14 ng/L	7/12 (58.3%)	2/3 (66.7%)	2/4 (50.0%)
NT-proBNP ≥ 300 ng/L	5/13 (38.5%)	1/5 (20.0%)	2/4 (50.0%)
Cardiac phenotype			
LVEF < 50% with VA	4 (16.0%)	2 (15.4%)	2 (28.6%)
High-grade AVB	9 (36.0%)	5 (38.5%)	2 (28.6%)
Echocardiogram			
LVEF (%)	60.0 (50.0–62.5)	60.0 (52.5–62.5)	52.5 (38.8–60.0)
LVEF < 50%	6 (24.0%)	3 (23.1%)	2 (28.6%)
Hypokinesia	9 (36.0%)	3 (23.1%)	3 (42.9%)
Akinesia	1 (4.0%)	0 (0.0%)	0 (0.0%)
IV septum thickness in diastole (mm)	9.1 (8.0–10.8); *n* = 22	9.0 (8.1–11.5); *n* = 11	8.2 (7.7–9.6); *n* = 6
Cardiac MRI			
LGE	17 (81.0%); *n* = 21	10 (90.9%); *n* = 11	3 (60.0%); *n* = 5
LV LGE: No. of segments involved	3.0 (1.0–5.5); *n* = 16	3.0 (1.0–5.0); *n* = 9	2.0 (1.5–9.5); *n* = 3
LV T2 hyperintensity (edema)	3/21 (14.3%)	1/11 (9.1%)	0/5 (0.0%)
RV LGE	5/21 (23.8%)	4/11 (36.4%)	0/5 (0.0%)
^18^F-FDG PET-CT			
FDG uptake pattern			
Cardiac alone	4/22 (18.2%)	0/10 (0.0%)	3 (42.9%)
Cardiac and mediastino-hilar LN	9/22 (40.9%)	7/10 (70.0%)	1 (14.3%)
Cardiac, lung, and mediastino-hilar LN	6/22 (27.3%)	3/10 (30.0%)	1 (14.3%)
LV FDG uptake: No. of segments involved	5.5 (2.0–8.0); *n* = 20	5.5 (2.5–6.8); *n* = 10	5.0 (2.2–7.8); *n* = 6
RV FDG uptake	7/22 (31.8%)	5/10 (50.0%)	0 (0.0%)

^18^F-FDG: fluorine-18 fluorodeoxyglucose; ACE: angiotensin-converting enzyme; AHA: American Heart Association; AVB: atrioventricular block; CKD-EPI: Chronic Kidney Disease Epidemiology Collaboration; CRP: C-reactive protein; CS: cardiac sarcoidosis; eGFR: estimated glomerular filtration rate; IQR: interquartile range; LGE: late gadolinium enhancement; LN: lymph nodes; LV: left ventricular; LVEF: left ventricular ejection fraction; MRI: magnetic resonance imaging; NT-proBNP: N-terminal pro-B-type natriuretic peptide; PET: positron emission tomography; RV: right ventricular; T2: T2-weighted MRI sequence.

**Table 3:** Immunosuppressive therapy, device implantation, and follow-up outcomes in the whole cohort, and stratified by relapse status.

Variables *n* (%) or Median (IQR)	Whole Cohort (*n* = 25)	Relapse Group (*n* = 13)	Non-Relapse Group (*n* = 7)
Immunosuppressive therapy			
IS therapy at diagnosis of CS	4 (16.0%)	1 (7.7%)	3 (42.9%)
IS therapy received before diagnosis of CS	7 (28.0%)	2 (15.4%)	4 (57.1%)
Time between first cardiac signs or symptoms and IS therapy initiation (months)	3.5 (1.0–7.0); *n* = 24	4.0 (1.0–7.0)	1.0 (1.0–6.0)
First line			
Prednisone	22 (88.0%)	12 (92.3%)	6 (85.7%)
Dosage (mg/kg/d)	0.60 (0.50–0.82); *n* = 22	0.60 (0.47–0.75); *n* = 12	0.68 (0.50–0.96); *n* = 6
Initial duration (months)	19.0 (11.0–26.5); *n* = 23	20.0 (11.1–23.8); *n* = 12	28.5 (20.2–38.2); *n* = 6
Second line			
Methotrexate SC	17 (68.0%)	10 (76.9%)	4 (57.1%)
Dosage (mg/kg/w)	0.22 (0.20–0.24); *n* = 18	0.22 (0.21–0.24); *n* = 10	0.20 (0.16–0.24); *n* = 5
Azathioprine	5 (20.0%)	2 (15.4%)	2 (28.6%)
Mycophenolate mofetil	2 (8.0%)	1 (7.7%)	1 (14.3%)
Second-line IS therapy duration (months)	24.0 (14.0–44.5); *n* = 24	24.0 (21.1–35.0)	52.0 (28.5–62.1)
Third line			
Infliximab	5 (20.0%)	4 (30.8%)	1 (14.3%)
Adalimumab	2 (8.0%)	1 (7.7%)	1 (14.3%)
Anti-arrhythmic	14 (56.0%)	8 (61.5%)	5 (71.4%)
Device implantation			
Pacemaker	7 (28.0%)	4 (30.8%)	1 (14.3%)
Defibrillator			
As initial device implantation	5 (20.0%)	2 (15.4%)	2 (28.6%)
As upgrade of an implanted device	5 (20.0%)	3 (23.1%)	1 (14.3%)
Resynchronization therapy			
As initial device implantation	3 (12.0%)	1 (7.7%)	1 (14.3%)
As upgrade of an implanted device	2 (8.0%)	1 (7.7%)	1 (14.3%)
**Outcomes and follow-up data**			
MACE	9 (36.0%)	5 (38.5%)	2 (28.6%)
PET Necrosis (follow-up studies)	7 (28.0%)	6 (46.2%)	1 (14.3%)
LVEF at last follow-up (%)	57.5 (50.0–62.5)	57.5 (50.0–62.5)	57.5 (55.0–61.0)

CS: cardiac sarcoidosis; IS: immunosuppressive; IQR: interquartile range; LVEF: left ventricular ejection fraction; MACE: major adverse cardiac events; SC: subcutaneous.

**Table 4:** Immunosuppressive therapy-related, device-related, and follow-up complications in patients with cardiac sarcoidosis.

Variables	*n* (%)
Corticosteroid-related	
Osteopenia	8/20 (40.0%)
Osteoporosis	4/20 (20.0%)
Weight gain	10/21 (47.6%)
Steroid-induced diabetes	4/22 (18.2%)
Steroid-induced hypertension	6/22 (27.3%)
Steroid-induced adrenal insufficiency	2/21 (9.5%)
Insomnia	7/21 (33.3%)
Nervosity	4/20 (20.0%)
Steroid-sparing agent-related	
Discontinuation of adverse events	7 (28.0%)
Prednisone	1 (4.0%)
Methotrexate	4 (16.0%)
Azathioprine	3 (12.0%)
Infections	
Multiple outpatient-treated infections	3/24 (12.5%)
Infection requiring hospitalization	5/24 (20.8%)
Newly diagnosed malignancy	2 (8.0%)
Device-related	
Inappropriate alarms	1/12 (8.3%)
Inappropriate shocks	2/12 (16.7%)
Device infection requiring removal	2/12 (16.7%)

**Table 5:** Number of patients and total events for each component of major adverse cardiac events.

Outcomes	No. of Patients	No. of Events
Cardiovascular hospitalizations	7	23
Defibrillator therapy	6	67 ^a^
Electrical storm	3	NA
LVAD implantation	0	0
Heart transplant	1	1
All-cause death	4	4
Cardiovascular-related death	2	2

LVAD: Left ventricular assist device. ^a^ This includes three patients with electrical storms (i.e., multiple appropriate shocks).

**Table 6:** Clinical presentation, treatment context, and imaging features of relapse episodes.

Variables *n* (%) or Median (IQR)	Relapse Group (*n* = 13)
Time from CS diagnosis to first relapse	36.0 (25.0–53.0)
Characteristics of relapses ^a^	
FDG uptake alone	8 (61.5%)
FDG uptake + arrhythmia	3 (23.0%)
FDG uptake + arrhythmia + symptoms	1 (7.7%)
Arrhythmia + symptoms	1 (7.7%)
FDG uptake (*n* = 12)	
Cardiac alone	3 (25.0%)
Cardiac and lymph nodes	2 (16.7%)
Cardiac, lymph nodes and lungs	6 (50.0%)
Lymph nodes alone	1 (8.3%)
LV uptake	11 (91.7%)
Number of segments involved	4.0 (2.5–5.5)
≥5 segments	5 (45.5%)
RV uptake	1 (8.3%)
Immunosuppressive therapy at relapse	8 (61.5%)
Prednisone	1 (7.7%)
Dose (mg/d)	10 (NA)
Methotrexate SC	6 (46.2%)
Dose (mg/kg/w)	0.15 (0.12–0.18)
Azathioprine	1 (7.7%)
Mycophenolate mofetil	1 (7.7%)
Infliximab	1 (7.7%)

CS: cardiac sarcoidosis; FDG: fluorodeoxyglucose; IQR: interquartile range; SC: subcutaneous. ^a^ Characteristics of relapses categorizes relapse presentations as follows: FDG uptake alone refers to asymptomatic relapse detected solely on follow-up PET-CT; FDG uptake + arrhythmia includes concurrent PET-CT abnormalities and new or worsening arrhythmias; FDG uptake + arrhythmia + symptoms includes all three components; and arrhythmia + symptoms refers to clinical relapse without PET-CT evidence.

**Table 7:** Comparison of prednisone regimens at diagnosis and at relapse among relapsing patients.

Variables	*n*	At Diagnosis Median (IQR)	At Relapse Median (IQR)	*p*-Value ^a^
Initial dose (mg/kg/day)	10	0.60 (0.43–0.60)	0.45 (0.26–0.50)	0.03
Time to <10 mg/day (months)	7	5.3 (4.3–6.2)	3.0 (2.8–3.3)	0.03
Total duration (months)	10	20.0 (11.5–22.5)	5.0 (5.0–9.8)	0.004

IQR: interquartile range. ^a^ Mann–Whitney U test.

## Data Availability

The original contributions presented in this study are included in the article/supplementary material. Further inquiries can be directed to the corresponding author.

## Supplementary Materials

The following supporting information can be downloaded at https://www.mdpi.com/article/10.3390/jcm14176234/s1: Table S1: Extracardiac organ involvement in the whole cohort, and stratified by relapse status; Table S2: Laboratory values at the diagnosis of cardiac sarcoidosis; Table S3: Prevalence of conduction and arrhythmic abnormalities on ECG, Holter, and device recordings in cardiac sarcoidosis patients; Table S4: Evolution of left ventricular ejection fraction from diagnosis to follow-up timepoints; Table S5: Comparison of clinical characteristics and imaging features at diagnosis of cardiac sarcoidosis between patients with and without MACE; and Table S6: Binary variables and sample size (n) considered for univariate Cox regression analysis of relapse and MACE in cardiac sarcoidosis.

## Abbreviations

The following abbreviations are used in this manuscript:

^18^F-FDG PET	^18^Fluor-fluorodeoxyglucose positron emission tomography
ACE	Angiotensin-converting enzyme
AHA	American Heart Association
AIVR	Accelerated idioventricular rhythm
ALAT	Alanine aminotransferase
AVB	Atrioventricular block
BMI	Body mass index
cMRI	Cardiac magnetic resonance imaging
CRP	C-reactive protein
CS	Cardiac sarcoidosis
DPI	Dossier Patient Informatisé
ECG	Electrocardiogram
ENT	Ear, nose, and throat
ESR	Erythrocyte sedimentation rate
FDG	Fluorodeoxyglucose
(a)HR	(adjusted) Hazard ratio
HRS	Heart Rhythm Society
ICD	Implantable cardioverter–defibrillator
IQR	Interquartile range
IS	Immunosuppressive
JCS	Japanese Circulation Society
LA	Left atrium
LGE	Late gadolinium enhancement
LN	Lymph node
LV	Left ventricle
LVAD	Left ventricular assist device
LVEF	Left ventricular ejection fraction
MACE	Major adverse cardiac event
MMF	Mycophenolate mofetil
MRI	Magnetic resonance imaging
MTX	Methotrexate
NRG	Non-relapse group
NT-proBNP	N-terminal pro B-type natriuretic peptide
PET	Positron emission tomography
PVC	Premature ventricular contraction
RA	Right atrium
RBBB	Right bundle branch block
RG	Relapse group
RV	Right ventricle
S6K	S6 kinase
SUVmax	Maximum standardized uptake value
T2	T2-weighted imaging
TNF	Tumor necrosis factor
VF	Ventricular fibrillation
VT	Ventricular tachycardia
