# Supplementary material for: Relapse Patterns and Clinical Outcomes in Cardiac Sarcoidosis: Insights from a Retrospective Single-Center Cohort Study
Source: J Clin Med. 2025 Sep 3;14(17):6234. doi: 10.3390/jcm14176234 (PMC12429488; doi:10.3390/jcm14176234)
Supplement: Supplementary file 1 [file jcm-14-06234-s001.zip › jcm-3820779-supplementary.pdf]

Supplementary Table S1: Extracardiac organ involvement in the whole cohort, and stratified by relapse status

Extracardiac involvement	Whole cohort (n = 25)	Relapse group (n = 13)	Non-relapse group (n = 7)
Lymph nodes	21 (84.0%)	12 (92.3%)	5 (71.4%)
Mediastino-hilar	21 (84.0%)	12 (92.3%)	5 (71.4%)
Axillar	4 (16.0%)	3 (23.1%)	0 (0.0%)
Cervical	4 (16.0%)	2 (15.4%)	0 (0.0%)
Infradiaphragmatic	5 (20.0%)	4 (30.8%)	0 (0.0%)
Lung	16 (64.0%)	7 (53.8%)	6 (85.7%)
ATS Stages			
0	3 (12.0%)	1 (7.7%)	1 (14.3%)
I	6 (24.0%)	5 (38.5%)	0 (0.0%)
II	13 (52.0%)	7 (53.8%)	4 (57.1%)
III	1 (4.0%)	0 (0.0%)	1 (14.3%)
IV	2 (8.0%)	0 (0.0%)	1 (14.3%)
Eyes	8 (32.0%)	4 (30.8%)	3 (42.9%)
Liver	3 (12.0%)	2 (15.4%)	0 (0.0%)
Spleen	1 (4.0%)	1 (7.7%)	0 (0.0%)
Skin	1 (4.0%)	1 (7.7%)	0 (0.0%)
Kidney	1 (4.0%)	0 (0.0%)	0 (0.0%)
Peripheral nervous system	2 (8.0%)	1 (7.7%)	1 (14.3%)
Arthritis	1 (4.0%)	1 (7.7%)	0 (0.0%)
Muscle	1 (4.0%)	0 (0.0%)	1 (14.3%)
Exocrine glands	2 (8.0%)	0 (0.0%)	1 (14.3%)
No. of organs involved	3 (3-4)	3 (3-4)	3 (3-4)
1	3 (12.0%)	1 (7.7%)	1 (14.3%)
2-3	14 (56.0%)	7 (53.8%)	4 (57.1%)
4-5	5 (20.0%)	4 (30.8%)	1 (14.3%)
≥6	3 (12.0%)	1 (7.7%)	1 (14.3%)

ATS: American Thoracic Society stages based on chest radiograph patterns of lung involvement.

Supplementary Table S2: Laboratory values at the diagnosis of cardiac sarcoidosis

Variables<br>*n* (%) or median (IQR)	Whole cohort<br>(*n* = 25)
Lymphocytes (10^9/l)	1.5 (1.1-1.7); *n* = 18
< 1.0 109/l	4/18 (22.2%)
ESR (mm/h)	12.0 (5.0-18.0); *n* = 19
> 20 mm/h	3/19 (15.8%)
CRP (mg/l)	2.3 (1.2-3.2); *n* = 20
> 5 mg/l	3/20 (15.0%)
Creatinine (μmol/l)	89.0 (73.0-96.0); *n* = 23
eGFR (CKD-EPI)	86.0 (74.0-92.0); *n* = 23
< 60 ml/min/1.73m^2	5/23 (21.7%)
ALAT (U/l)	29.0 (22.5-38.5); *n* = 22
> 60 U/l	1/22 (4.5%)
Angiotensin-converting enzyme (U/l)	
> 70 U/l	4/20 (20.0%)
Calcitriol (pmol/l);	103.0 (59.0–115.4); n = 17
Calcidiol (nmol/l)	62.0 (32.0–72.5); n = 16
Calcitriol/calcidiol ratio	1.6 (1.3-2.8); n = 14
Corrected calcium (mmol/l)	2.3 (2.2-2.3); n = 23
> 2.52 mmol/L	1/23 (4.3%)
sIL-2R (ng/ml)	
⩾ 2 ng/ml	2/6 (33.3%)
High-sensitive troponin T (ng/l)	15.5 (7.5-32.2); n = 12
⩾ 14 ng/l	7/12 (58.3%)
NT-proBNP (ng/l)	207.0 (80.0–1012.0); n=13
⩾ 300 ng/l	5/13 (38.5%)
IgG (g/l)	11.9 (10.5–15.5); n=14
< 7 g/l	1/14 (7.1%)
> 16 g/l	3/14 (21.4%)

ALAT: alanine aminotransferase; CKD-EPI: Chronic Kidney Disease Epidemiology Collaboration; CRP: C-reactive protein; eGFR: estimated glomerular filtration rate; ESR: erythrocyte sedimentation rate; IgG: immunoglobulin G; NT-proBNP: N-terminal pro–B-type natriuretic peptide; sIL-2R: soluble interleukin-2 receptor.

Supplementary Table S3: Prevalence of conduction and arrhythmic abnormalities on ECG, Holter, and device recordings in cardiac sarcoidosis patients

Variables n (%)	ECG at diagnosis (*n* = 24)	Holter at diagnosis (*n* = 22)	All recordings during follow-up (*n* = 23)	Recording at last follow-up (*n* = 18)
No abnormalities	8 (33.3%)	2 (9.1%)	0 (0.0%)	4 (22.2%)
Atrioventricular conduction abnormalities				
1st degree AVB	5 (20.8%)	4 (18.2%)	10 (43.5%)	3 (16.7%)
2nd degree AVB Mobitz 1	0 (0.0%)	1 (4.5%)	3 (13.0%)	2 (11.1%)
2nd degree AVB Mobitz 2	0 (0.0%)	0 (0.0%)	0 (0.0%)	0 (0.0%)
2nd degree AVB 2:1	0 (0.0%)	1 (4.5%)	2 (8.7%)	0 (0.0%)
3rd degree AVB	4 (16.7%)	4 (18.2%)	7 (30.4%)	3 (16.7%)
Supraventricular arrhythmias				
AVNRT	0 (0.0%)	1 (4.5%)	1 (4.3%)	0 (0.0%)
AVRT	0 (0.0%)	1 (4.5%)	1 (4.3%)	0 (0.0%)
PAC ⩾1%	0 (0.0%)	0 (0.0%)	2 (8.7%)	1 (5.6%)
Atrial tachycardia	0 (0.0%)	3 (13.6%)	8 (34.8%)	1 (5.6%)
Atrial fibrillation	0 (0.0%)	1 (4.5%)	4 (17.4%)	1 (5.6%)
Atrial flutter	0 (0.0%)	1 (4.5%)	3 (13.0%)	0 (0.0%)
Ventricular arrhythmias				
PVC	3 (12.5%)	NA	NA	NA
PVC <1%	NA	3 (13.6%)	7 (30.4%)	3 (16.7%)
PVC 1–5%	NA	1 (4.5%)	6 (26.1%)	0 (0.0%)
PVC 5.1–10%	NA	1 (4.5%)	7 (30.4%)	2 (11.1%)
PVC 10.1–20%	NA	3 (13.6%)	7 (30.4%)	0 (0.0%)
PVC >20%	NA	2 (9.1%)	5 (21.7%)	0 (0.0%)
nsVT	0 (0.0%)	6 (27.3%)	12 (52.2%)	1 (5.6%)
AIVR	0 (0.0%)	3 (13.6%)	8 (34.8%)	0 (0.0%)
VT	1 (4.2%)	3 (13.6%)	9 (39.1%)	0 (0.0%)
VF	0 (0.0%)	0 (0.0%)	1 (4.3%)	0 (0.0%)
Electrical Storm	0 (0.0%)	0 (0.0%)	3 (13.0%)	0 (0.0%)
Intraventricular conduction abnormalities				
RBBB	5 (20.8%)	3 (13.6%)	6 (26.1%)	0 (0.0%)
LBBB	1 (4.2%)	2 (9.1%)	3 (13.0%)	1 (5.6%)
LAFB	3 (12.5%)	0 (0.0%)	4 (17.4%)	0 (0.0%)
LPFB	0 (0.0%)	0 (0.0%)	0 (0.0%)	0 (0.0%)
Sinus node abnormalities				
Sinus Tachycardia	1 (4.2%)	0 (0.0%)	1 (4.3%)	0 (0.0%)
Sinus Bradycardia	2 (8.3%)	1 (4.5%)	3 (13.0%)	0 (0.0%)
Sinus pause ⩾ 2–⩽ 3 sec	0 (0.0%)	1 (4.5%)	2 (8.7%)	0 (0.0%)
Sinus arrest > 3sec	0 (0.0%)	0 (0.0%)	1 (4.3%)	2 (11.1%)
Chronotropic incompetence	0 (0.0%)	0 (0.0%)	0 (0.0%)	0 (0.0%)
Sick sinus syndrome	0 (0.0%)	0 (0.0%)	1 (4.3%)	2 (11.1%)

AIVR: accelerated idioventricular rhythm; AVB: atrioventricular block; AVNRT: atrioventricular nodal reentrant tachycardia; AVRT: atrioventricular reentrant tachycardia; LAFB: left anterior fascicular block; LBBB: left bundle branch block; LPFB: left posterior fascicular block; nsVT: non-sustained ventricular tachycardia; PAC: premature atrial contraction; PVC: premature ventricular contraction; RBBB: right bundle branch block; VF: ventricular fibrillation; VT: ventricular tachycardia.

Supplementary Table S4: Evolution of left ventricular ejection fraction from diagnosis to follow-up timepoints

Timepoint	*n*	LVEF at diagnosis median (IQR)	LVEF at each timepoint median (IQR)	p-Value[a]
**6 months**	16	61.3 (52.5–62.5)	58.3 (51.9–62.5)	0.55
**12 months**	15	57.5 (47.5–62.5)	58.0 (53.8–62.5)	0.63
**24 months**	14	57.5 (44.4–62.5)	55.0 (50.5–61.9)	0.64
**60 months**	10	61.3 (53.8–62.5)	60.0 (55.6–64.4)	0.67
**Last follow-up**	22	60.0 (52.5–62.5)	60.0 (55.0–62.5)	0.77

LVEF: left ventricular ejection fraction; IQR: interquartile range.

[a] Mann–Whitney U test.

Supplementary Table S5: Comparison of clinical characteristics and imaging features at diagnosis of cardiac sarcoidosis between patients with and without MACE

Variables<br>*n* (%) or median (IQR)	Without MACE<br>(*n* = 16)	With MACE<br>(*n* = 9)	*p*-Value[a]
**Demographic characteristics and comorbidities**			
Age at diagnosis of CS (years)	52.5 (47.8–61.8)	55.0 (47.0–60.0)	
Follow up since diagnosis of CS (months)	58.5 (49.2–64.3)	54.0 (27.5–99.0)	
Arrhythmia considered idiopathic before diagnosis of CS	1 (6.2%)	3 (33.3%)	
Time between idiopathic arrhythmia and diagnosis of CS (months)	85.1 (NA); n=1	108.0 (71.5–120.1); n=3	
Cardiac relapse	9 (56.3%)	5 (55.6%)	
Extra-cardiac relapse	2 (12.5%)	1 (11.1%)	
Male	14 (87.5%)	5 (55.6%)	
Caucasian	15 (93.8%)	8 (88.9%)	
Smoking habit			
Never	10 (62.5%)	6 (66.7%)	
Former	5 (31.2%)	3 (33.3%)	
BMI (kg/m²)	26.6 (25.1–33.2)	25.8 (25.2–26.1)	
Comorbidities			
Dyslipidemia	7 (43.8%)	2 (22.2%)	
Diabetes	1 (6.2%)	1 (11.1%)	
Hypertension	8 (50.0%)	3 (33.3%)	
Sleep Apnea Syndrome	4 (25.0%)	1 (11.1%)	
Ischemic cardiomyopathy	1 (6.2%)	0 (0.0%)	
Chronic Kidney Insufficiency	3 (18.8%)	2 (22.2%)	
Associated autoimmune disease	4 (25.0%)	1 (11.1%)	
Diagnostic criteria - AHA likelihood algorithm[11]			
Definite	0 (0.0%)	4 (44.4%)	0.01
Highly probable	5 (31.2%)	3 (33.3%)	
Probable	11 (68.8%)	2 (22.2%)	
**Initial presentation of cardiac sarcoidosis**			
CS diagnosed as initial sarcoidosis presentation	8 (50.0%)	5 (55.6%)	
Symptoms			
Palpitations	5 (31.2%)	5 (55.6%)	
Syncope	2 (12.5%)	0 (0.0%)	
Dizziness	4 (25.0%)	2 (22.2%)	
Chest pain	2 (12.5%)	2 (22.2%)	
Shortness of breath	6 (37.5%)	5 (55.6%)	
Clinical heart failure	0 (0.0%)	4 (44.4%)	0.01
No cardiac symptoms	3 (18.8%)	1 (11.1%)	
Laboratory values			
Lymphocytes <1.5 10^9/l	7/13 (53.8%)	2/5 (40.0%)	
CRP >5 mg/l	2/13 (15.4%)	1/7 (14.3%)	

ACE >70 U/l	3/10 (30.0%)	1/6 (16.7%)	
High-sensitive troponin T ⩾14 ng/l	2/7 (28.6%)	5/5 (100.0%)	0.03
NT-proBNP ⩾300 ng/l	1/9 (11.1%)	4/4 (100.0%)	0.01
Cardiac phenotype			
LVEF<50% with ventricular arrhythmia	0 (0.0%)	4 (44.4%)	0.01
High-grade AVB	5 (31.2%)	4 (44.4%)	
Echocardiogram			
LVEF at diagnosis (%)	62.5 (56.2–62.5)	37.5 (27.5–60.0)	0.02
LVEF <50%	1 (6.2%)	5 (55.6%)	0.01
Cardiac MRI			
LGE	5 (71.4%); n=7	1 (25.0%); n=4	
LV LGE: No. of segments involved	2.0 (1.0–3.5); n=11	5.0 (5.0–8.0); n=5	0.03
RV LGE	3 (18.8%)	2 (22.2%)	
18F-FDG PET-CT			
LV FDG: No. of segments involved	3.5 (2.0–6.8); n=14	7.5 (6.2–11.8); n=6	
RV FDG	0 (0.0%)	4 (44.4%)	0.01
PET-CT necrosis (at diagnosis or during follow up)	2 (12.5%)	5 (55.6%)	
**Management**			
IS therapy at diagnosis of CS	3 (18.8%)	1 (11.1%)	
Time between first cardiac signs or symptoms and IS therapy initiation (months)	3.0 (1.0-6.5); n=15	4.0 (1.0–7.0)	
Prednisone dosage at diagnosis of CS	0.50 (0.50–0.75); n=13	0.75 (0.60–1.00)	
Duration of initial treatment (months)	18.0 (11.1–21.0); n=13	26.1 (10.0–41.0)	
Defibrillator implantation	3 (18.8%)	7 (77.8%)	0.01
Resynchronization therapy	0 (0.0%)	5 (55.6%)	0.002

ACE: angiotensin-converting enzyme; AVB: atrioventricular block; BMI: body mass index; CRP: C-reactive protein; CS: cardiac sarcoidosis; FDG: fluorodeoxyglucose; IS: immunosuppressive; IQR: interquartile range; LGE: late gadolinium enhancement; LV: left ventricular; LVEF: left ventricular ejection fraction; MRI: magnetic resonance imaging; NT-proBNP: N-terminal pro–B-type natriuretic peptide; PET-CT: positron emission tomography-computed tomography; RV: right ventricular.

[a] Categorical variables were compared using Fisher’s exact test. Continuous variables were compared using the Mann–Whitney U test.

Supplementary Table S6: Binary variables and sample size (n) considered for univariate Cox regression analysis of relapse and MACE in cardiac sarcoidosis

Variables	*n*
Age ⩾53 years old	25
Female	25
Smoking status: never	25
BMI ⩾26 kg/m^2	25
Hypertension	25
Diabetes	25
Chronic Kidney Insufficiency	25
Associated autoimmune disease	25
AHA Diagnostic criteria: Definite	25
Isolated cardiac sarcoidosis	25
No. of organs involved ⩾4	25
Lung involvement	25
Eye involvement	25
Liver involvement	25
Lymphocytes <1.5 x10^9/l	18
CRP >5 mg/l	20
eGFR (CKD-EPI) <60 ml/min/1.73m^2	23
ACE >70 U/l	16
hs-Troponin T ⩾14 ng/l	12
NT-proBNP ⩾300 ng/l	13
Ventricular arrhythmias	25
High-grade AVB	25
LVEF <50% with ventricular arrhythmias	25
LVEF <50%	25
Hypokinesia	25
LV FDG uptake ⩾5 segments	20
LV necrosis	25
RV FDG uptake	22
LV LGE ⩾5 segments	16
Time to IS therapy initiation ⩾3.5 months	24
First episode prednisone duration ⩽18 months	22
First episode second-line IS therapy ⩽24months	23
Anti-TNF therapy	25
MACE	25
Relapse	25

ACE: angiotensin-converting enzyme; AHA: American Heart Association; AVB: atrioventricular block; BMI: body mass index; CKD-EPI: Chronic Kidney Disease Epidemiology Collaboration; CRP: C-reactive protein; eGFR: estimated glomerular filtration rate; FDG: fluorodeoxyglucose; hs-Troponin T: high-sensitivity troponin T; IS: immunosuppression; LGE: late gadolinium enhancement; LVEF: left ventricular ejection fraction; LV: left ventricle; MACE: major adverse cardiac events; NT-proBNP: N-terminal pro–B-type natriuretic peptide; RV: right ventricle; TNF: tumor necrosis factor.
